# FasL −844T/C and Fas −1377G/A: mutations of pulmonary adenocarcinoma in South China and their clinical significances

**DOI:** 10.1007/s13277-015-3071-5

**Published:** 2015-01-18

**Authors:** Hongguang Zhao, Wenhu Chen, Peng Du, Aihua Sun, Chenyu Zhuang, Jiali Tong, Lifang Wang

**Affiliations:** 1 Department of Thoracic Surgery, Zhejiang Key Laboratory of Diagnosis and Treatment Technology on Thoracic Oncology (Lung and Esophagus), Zhejiang Cancer Hospital, Hangzhou, 310022 China; 2 Department of Biochemistry, Institute of Basic Medical Science, Zhejiang Medical College, Hangzhou, 310053 China; 3 Class 1201/1202, Department of Clinical Medicine, Zhejiang Medicine College, Hangzhou, 310053 China

**Keywords:** FasL, Fas, SNP, Pulmonary adenocarcinoma

## Abstract

Apoptosis is an important mechanism of malignant tumor formation and progression. Single nucleotide polymorphisms (SNPs) located within cell death genes may influence cancer risk. We explored the relationship between FasL −844T/C and/or Fas −1377G/A SNPs and pulmonary adenocarcinoma (AD). Two hundred seventy-five patients with pulmonary AD of South China admitted into Zhejiang Cancer Hospital from July 2007 to October 2011 were randomly selected, and their clinicopathological data were collected at the same time. Two hundred ninety-seven cases of healthy individuals were selected as control. FasL −844T/C and Fas −1377G/A SNPs were detected by PCR-RFLP technique to evaluate the relationships between these two SNPs and pulmonary AD. Age, FasL −844 and Fas −1377 SNPs were associated with increased risk of pulmonary AD susceptibility in main effect analysis. FasL −844CC and Fas −1377 AA were associated with an increased risk for the development of pulmonary AD only in age <60 years people, but not in those ≥60 years. FasL −844CC genotype was associated with an increased risk for pulmonary AD (adjusted OR = 2.010, 95 % CI 1.196–3.379, *P* = 0.008) compared with TT genotype. However, Fas −1377 AA was a risk factor only when FasL −844 genotype was CC. Fas −1377 genotypes showed significant effect modification of pulmonary AD risk by FasL −844 genotype with test of the interaction term adjusting for age, gender, and FasL −844 SNP. Fas −1377G/A was not associated with the clinicopathological factors, while FasL −844C/T was associated with tumor stage and lymph node metastasis in age ≥60 years people and tumor stage in those <60 years. In conclusion, FasL −844 SNP is associated with the susceptibility of pulmonary AD in age <60 years people. Fas −1377 SNP may modify the association of FasL −844 SNP with the risk of pulmonary AD. FasL −844 genotype plays an important role in the occurrence and progression of pulmonary AD.

## Introduction

Lung cancer is the most common primary malignancy and the leading cause of cancer-related deaths worldwide [1]. Non-small cell lung cancer (NSCLC) accounts for 85 % of all lung cancers. Adenocarcinoma (AD) accounts for 50 % of NSCLC cases. It is one of the most frequent histologic subtypes and presently the most common type in most countries. Since 30–40 years ago, the incidence of AD has increased steadily [2, 3].

Approximately 80–90 % of lung carcinomas are associated with the carcinogenetic effect of smoking [4]. However, only about 10–15 % of all smokers fall ill on lung cancer. On the other side, 10–15 % of all lung carcinomas occur in nonsmokers [5]. These facts indicate that there is individual susceptibility to lung carcinogens as well as to development of disorders. Genetic and environmental factors essentially influence the risk of lung cancer genesis, while molecular pathophysiology of genetic-environmental interaction is complex and only partly identified up to now. That is why it is so important to explore, whether individual difference in susceptibility to lung cancer genesis has a genetic background.

Acquired ability to resist apoptosis is a common hallmark of various types of malignant diseases, and the regulatory defects of components of the apoptosis pathway contribute to tumorigenesis, tumor cell invasion, and metastasis [6, 7]. Genetic variations of crucial genes in this cell-death pathway may thus influence the susceptibility to cancer. The Fas–Fas ligand (FasL) system is recognized as a major pathway for the induction of apoptosis in cells and tissues [8]. Fas interacts with FASL to initiate the death signal cascade and activate the apoptosis signaling pathway. Fas and FasL molecules play an important role in immune escape [9]. Decreased expression of Fas and/or increased expression of FasL is associated with different malignancies and may favor malignant transformation and progression [10].

Single nucleotide polymorphisms (SNPs) have been proposed to play an important role in the genetic susceptibility to cancer. Many gene SNPs can impact the risk of cancer. The functional mutations in the Fas/FasL genes that impair apoptotic signal transduction have been shown to be associated with an increased risk of many types of cancers [11–15]. The Fas/FasL system may be significant in cancer initiation, development, and progression, and SNPs, which possess the potential to alter the expression of Fas and/or FasL, have been proposed to be significant in the genetic susceptibility to cancer.

We have studied the clinical significances of FasL −844T/C and Fas −1377G/A mutation in esophageal carcinoma [16]. Now, we want to analyze whether FasL −844T/C and Fas −1377G/A polymorphism influence the susceptibility or clinicopathological factor of pulmonary AD.

## Materials and methods

### Subjects

Patients with histopathologically confirmed pulmonary AD (*n* = 275) were recruited from Zhejiang Cancer Hospital, affiliated hospital of Zhejiang Medical College, China, between July 2007 and October 2011. Control subjects (*n* = 297) were cancer-free individuals who had been randomly selected from medical examination center. All subjects were unrelated ethnic Han Chinese and residents in South China. There was no sex and age restriction. Before recruitment, a standard questionnaire had been administered through face-to-face interviews by trained interviewers to obtain information on demographic data and related factors. At recruitment, written informed consent was obtained from each subject to consent to participate in the study and to allow their biological samples to be genetically analyzed. This research was approved by the Medical Ethics Committee, Zhejiang Cancer Hospital.

All patients underwent radical resection and had detailed clinicopathological data. The tumor staging was determined according to UICC 2009 7th Edition. The presence or absence of lymph node metastasis was evaluated according to the tumor node metastasis classification on the basis of postoperative histopathologic examination of pulmonary AD specimens. Other clinical data (age at diagnosis, sex, tumor length) and tumor biological data (grading, visceral pleura, vascular tumor thrombus) were also documented.

### Genotyping

After signing informed consent forms, each subject donated 3 mL of blood to be used for genomic DNA extraction.

Genomic DNA was extracted from peripheral blood using Blood Genome DNA Extraction Kit (Takara Biotechnology Co. Ltd., Dalian). The −844T/C polymorphism of FasL was detected using PCR-RFLP. DNA fragments were amplified in a total volume of 100 μL PCRs containing PCR Premix 50 μL (BioTeke Corporation, Beijing, China), forward primer (5′-CAGCTACTCGG AGGCCAAG-3′) 1 μL, reverse primer (5′-GCTCTGAGGGGAGAGACCAT-3′) 1 μL, DDW 46 μL, and DNA 2 μL. Amplification was carried out under the following condition: 1 cycle of 2 min at 95 °C, 35 cycles of 30 s at 94 °C, 30 s at 62 °C, and 45 s at 72 °C, followed by 7 min at 72 °C. Amplified products were digested with BsrDI (Fermentas, Thermo Fisher Scientific Inc.) at 55 °C for 4 h. All products were loaded onto 3 % MS-6 Agarose (Takara Biotechnology Co. Ltd., Dalian) and electrophoresed. Bands were visualized and typed after GelRed (Biotium Company, USA) staining. The PCR product amplified for the polymorphism was 410 bp. The BsrDI restriction enzymes were used to distinguish −844T/C, resulting in 122- and 104-bp fragments in the presence of the −844T allele.

Fas −1377G/A polymorphism in promoter region was also detected with PCR-RFLP. The primers for amplification were 5′-TGTGTGCACAAGGCTGGCGC-3′ for forward primer and 5′-TGCATCTGTCACTGCACTTACCACCA-3′ for reverse primer, which produce 122-bp fragment. To introduce a restriction endonuclease site, 3′ end of forward primer was changed from CAC to CGC, which created a BstUI site. The procedure of amplification was the same as above. After PCR, BstUI (New England Biolabs, USA) digestion generated fragments of 104 and 18 bp for G allele.

The polymorphism analysis was performed by two persons independently in a blind fashion. More than 10 % of the samples were randomly selected for confirmation, and the results were 100 % concordant.

### Statistical analyses

Values were expressed as mean ± SD or percent. To check for genotyping error, we examined departure from Hardy–Weinberg equilibrium (HWE) in controls, using *χ* ^2^ test. *χ* ^2^ test analysis was used to detect these SNPs in patients with pulmonary AD and health individuals. The *t* test and *χ* ^2^ test were used to detect age and sex in the two groups, respectively.

Association between FasL −844T/C or Fas −1377G/A polymorphism and pulmonary AD was assessed using *χ* ^2^ test. Pulmonary AD risk was estimated by odds ratios (OR) and 95 % confidence intervals (CI) using conditional logistic regression or multinomial logistic regression controlling for age and gender. This codominant model was defined as heterozygotes (1 variant genotype) versus wild-type (0 variant genotype) or homozygotes (2 variant genotype) versus wild-type.

The relationships of these SNPs with the clinicopathological parameters of patients were analyzed by *χ* ^2^ test. We used binary logistic regression (Ascendant Wald method) with each parameter as dependant variable and genotypes of FasL −844T/C or Fas −1377G/A polymorphism as independent variables. Clinicopathological parameters were dichotomized as follows: grading (well and moderately differentiation versus poorly differentiation), stage (stage I versus stages II–IIIa), tumor length (less than and equal to 3 cm versus greater than 3 cm), visceral pleura, lymph node metastasis, and vascular tumor thrombus (positive versus negative).

For stratified analyses, we created indicator variable of age greater or less than 60 years old. Then, the above statistical analyses were carried out at different age groups.

All statistical testing was done at the two-sided 0.05 level with SPSS 16.0 software.

## Results

### Gene polymorphisms and the association with pulmonary AD

This population-based case-control study included 275 patients with pulmonary AD and 297 control subjects. All study subjects were south Chinese. Baseline clinical characteristics, the genotype, and allele distributions of FasL and Fas polymorphisms in cases and controls are summarized in Table 1.

**Table 1:** Baseline clinical characteristics and the genotypic and allelic frequencies of SNPs of cases and controls

		Cases (%) (*n* = 275)	Controls (%) (*n* = 297)	*χ* ^2^	*P* value
Gender	male	147	179	2.705	0.100
	female	128	118		
Age	<60	119	192	26.293	0.000**
	≥60	156	105		
Smoking status	no	219	254	0.062	0.803
	yes	56	63		
FasL −844 Genotypes	TT	32 (11.6)	52 (17.5)	8.692	0.013*
	TC	103 (37.5)	128 (43.1)		
	CC	140 (50.9)	117 (39.4)		
Alleles	T allele	167 (30.4)	232 (39.1)	9.503	0.002**
	C allele	383 (69.6)	362 (60.9)		
Fas −1377 Genotypes	GG	89 (32.4)	121 (40.7)	6.605	0.037*
	GA	133 (48.4)	138 (46.5)		
	AA	53 (19.3)	38 (12.8)		
Alleles	G allele	311 (56.5)	380 (64.0)	6.587	0.010*
	A allele	239 (43.5)	214 (36.0)		

*P < 0.05; **P < 0.01

Cases were older than controls (*P* = 0.000). The FasL −844T/C and Fas −1377G/A genotype frequencies were all in agreement with Hardy–Weinberg equilibrium in the controls (*χ* ^2^ = 2.663, *P* = 0.103; *χ* ^2^ = 0.019, *P* = 0.890, respectively).

Age, FasL −844, and Fas −1377 SNPs were associated with increased risk of pulmonary AD susceptibility in main effect analysis (Table 2).

**Table 2:** Main effects of individual risk factors on pulmonary AD risk by Logistic regression

Variable	Crude OR (95 % CI)	*P* value	Adjusted OR (95 % CI)	*P* value
Age	2.397 (1.712–3.357)	0.000	2.610 (1.815–3.754)^a^	0.000
FasL −844 locus	1.418 (1.121–1.794)	0.004	1.418 (1.111–1.811)^b^	0.005
Fas −1377 locus	1.363 (1.074–1.730)	0.011	1.364 (1.065–1.746)^c^	0.014

^a^Adjusting for gender, smoking status, FasL −844, and Fas −1377 SNP

^b^Adjusting for age, gender, smoking status, and Fas −1377 SNP

^c^Adjusting for age, gender, smoking status, and FasL −844 SNP

### Effect modification of the associations of the SNP with pulmonary AD

Logistic regression analysis was used to estimate associations between the genotypes and risk of pulmonary AD (Table 3). Adjusting for age and gender, FasL −844CC genotype was associated with an increased risk for pulmonary AD (aOR = 2.010, 95 % CI = 1.196–3.379, *P* = 0.008) compared with TT genotype. The C haplotype remained a significant risk for pulmonary AD (aOR = 1.504, 95 % CI = 1.169–1.936, *P* = 0.002), compared with T haplotype. Fas −1377 AA genotype was also associated with an increased risk for pulmonary AD (aOR = 1.400, 95 % CI 1.083–1.809, *P* = 0.010) compared with GG genotype.

**Table 3:** Association of genotypic and allelic frequencies of FasL −844 and Fas −1377 with pulmonary AD risk

Locus		Crude OR (95 % CI)	*P* value	Adjusted OR^a^ (95 % CI)	*P* value
FasL −844	Genotypes				
	TT	1		1	
	TC	1.308 (0.784–2.180)	0.304	1.300 (0.769–2.198)	0.328
	CC	1.944 (1.174–3.219)	0.010*	2.010 (1.196–3.379)	0.008**
	Alleles				
	T allele	1		1	
	C allele	1.470 (1.150–1.7879)	0.002**	1.504 (1.169–1.936)	0.002**
Fas −1377	Genotypes				
	GG	1		1	
	GA	1.310 (0.912–1.883)	0.144	1.252 (0.860–1.823)	0.241
	AA	1.377 (1.073–1.767)	0.012*	1.400 (1.083–1.809)	0.010*
	Alleles				
	G allele	1		1	
	A allele	1.233 (0.938–1.620)	0.133	1.242 (0.937–1.646)	0.132

*P < 0.05; **P < 0.01

^a^Adjusting for age and gender

Risk of pulmonary AD was evaluated by combining FasL −844 genotypes and Fas −1377 genotypes (Table 4). FasL −844CC was still a risk factor for pulmonary AD no matter what Fas −1377 genotypes was. However, Fas −1377 AA was not a risk factor for pulmonary AD when FasL −844 genotype was TT and TC but was a risk factor when FasL −844 genotype was CC.

**Table 4:** Risk of pulmonary AD associated with FasL −844 genotypes by Fas −1377 genotypes

FasL −844	Fas −1377	Cases (%) (*n* = 275)	Controls (%) (*n* = 297)	Crude OR (95 % CI)	*P* value	Adjusted OR^a^ (95 % CI)	*P* value
TT + TC	GG + GA	113	156	1		1	
TT + TC	AA	22	24	1.265 (0.676–2.369)	0.462	1.442 (0.750–2.774)	0.273
CC	GG + GA	109	103	1.209 (1.009–1.449)	0.04	1.228 (1.016–1.484)	0.033
CC	AA	31	14	1.451 (1.159–1.818)	0.01	1.519 (1.201–1.922)	0.000
CC	GG + GA	109	103	1		1	
CC	AA	31	14	2.092 (1.054–4.156)	0.035	2.108 (1.025–4.337)	0.043

^a^Adjusting for age and gender

Fas −1377 genotypes showed significant effect modification of pulmonary AD risk by FasL −844 genotype by test of the interaction term adjusting for age, gender, FasL −844, and Fas −1377 SNP (aOR = 1.531, 95 % CI = 1.066–2.201, *P* = 0.021).

### The relationships between clinicopathological significance and the polymorphisms

The relationships between pulmonary AD clinicopathological parameters and these two SNPs were compared (Table 5). There were no significant differences between Fas −1377 gene polymorphism and tumor size, differentiation visceral pleura, stage, lymph node metastasis, and vascular tumor thrombus, while FasL −844C/T was associated with tumor stage and lymph node metastasis. CC versus TT was dangerous, aOR = 2.273 (1.436–3.599), *P* = 0.000; aOR = 2.053 (1.297–3.249), and *P* = 0.002, respectively (Table 6).

**Table 5:** Relationships between pulmonary AD clinicopathological parameters and FasL −844T/C or Fas −1377G/A SNPs

Number (%)		Tumor length		Differentiation		Visceral pleura		Stage		Lymph node metastasis		Vascular tumor thrombus	
Locus	Genotypes	≤3 cm	>3 cm	Well moderately	Poorly moderately	Yes	No	I	II + IIIa	Yes	No	Yes	No
FasL −844	TT	20 (7.3)	12 (4.4)	15 (5.5)	17 (6.2)	12 (4.7)	20 (7.3)	25 (9.1)	7 (2.5)	25 (9.1)	7 (2.5)	27 (9.8)	5 (1.8)
	TC	56 (20.4)	47 (17.1)	55 (20.0)	48 (17.5)	35 (12.7)	68 (24.7)	59 (21.5)	44 (16.0)	63 (22.9)	40 (14.5)	91 (33.1)	12 (4.4)
	CC	71 (25.8)	69 (25.1)	75 (27.3)	65 (23.6)	42 (15.3)	98 (35.6)	59 (21.5)	81 (29.5)	67 (24.4)	73 (26.5)	115 (41.8)	25 (9.1)
*χ* ^2^		1.509		0.498		0.866		15.353		11.246		1.77	
*P* value		0.470		0.779		0.649		0.000^*^		0.004*		0.413	
Fas −1377	GG	47 (17.1)	42 (15.3)	51 (18.5)	38 (13.8)	30 (10.9)	59 (21.5)	49 (17.8)	40 (14.5)	52 (18.9)	37 (13.5)	79 (28.7)	10 (3.6)
	GA	67 (24.4)	66 (24.0)	64 (23.3)	69 (25.1)	40 (14.5)	93 (33.8)	70 (25.5)	63 (22.9)	75 (27.3)	58 (21.1)	109 (39.6)	24 (8.7)
	AA	33 (12.0)	20 (7.3)	30 (10.9)	23 (8.4)	19 (6.9)	34 (12.4)	24 (8.7)	29 (10.5)	28 (29.1)	25 (9.1)	45 (16.4)	8 (2.9)
*χ* ^2^		2.175		2.2		0.686		1.312		0.423		1.912	
*P* value		0.337		0.333		0.71		0.519		0.809		0.384	

*P < 0.05; **P < 0.01

**Table 6:** Risk of pulmonary adenocarcinoma stage and lymph node metastasis according to FasL −844 genotypes

Locus	Genotypes	Stage		Lymph node metastasis	
		Adjusted OR^#^ (95 % CI)	*P*	Adjusted OR^#^ (95 % CI)	*P*
FasL −844	TT	1		1	
	TC	2.573 (1.003–6.601)	0.049*	2.163 (0.844–5.544)	0.108
	CC	2.273 (1.436–3.599)	0.000**	2.053 (1.297–3.249)	0.002**

*P < 0.05; **P < 0.01

^#^Adjusting for age and gender

### Association of the polymorphisms and pulmonary AD stratified by age

Patients with pulmonary AD and healthy group were stratified by age to explore whether FasL −844T/C and Fas −1377G/A polymorphisms were associated with pulmonary AD in this selected population of patients (Table 7). The two genotype frequencies in two control subgroups (<60 and ≥60 years old) agreed with frequencies expected under the Hardy–Weinberg equilibrium (*χ* ^2^ = 1.852, *P* = 0.174 and *χ* ^2^ = 0.792, *P* = 0.373 for FasL −844C/T; *χ* ^2^ = 0.022, *P* = 0.881 and *χ* ^2^ = 0.197, *P* = 0.657 for Fas −1377G/A, respectively).

**Table 7:** Genotypic and allelic frequencies of FasL −844 and Fas −1377 in subgroups according to age

Number (%)	Age	Smoking status		Gender		FasL −844 genotypes			Fas −1377 genotypes		
	<60	No	Yes	Male	Female	TT	TC	CC	GG	GA	AA
Cases (*n* = 119)		109 (91.6)	10 (8.4)	62 (52.1)	57 (47.9)	12 (10.1)	43 (36.1)	64 (53.8)	41 (34.5)	48 (40.3)	30 (25.2)
Controls (*n* = 192)		170 (88.5)	22 (11.5)	114 (59.4)	78 (40.6)	35 (18.3)	83 (43.2)	74 (38.5)	78 (40.6)	88 (45.8)	26 (13.5)
*χ* ^2^			0.74		1.582			7.983			6.794
*P*			0.39		0.208			0.018^*^			0.033^*^
	≥60										
Cases (*n* = 156)		110 (70.5)	46 (29.5)	85 (54.5)	71 (45.5)	20 (12.8)	60 (38.5)	76 (48.7)	48 (30.8)	85 (54.5)	23 (14.7)
Controls (*n* = 105)		64 (61.0)	41 (39.0)	65 (61.9)	40 (38.1)	17 (16.2)	45 (42.9)	43 (41.0)	43 (41.0)	50 (47.6)	12 (11.4)
*χ* ^2^			2.58		1.413			1.634			2.953
*P*			0.11		0.235			0.442			0.228

*P < 0.05; **P < 0.01

With age <60 years, the frequencies of the TT, TC, and CC of FasL −844 genotypes were 10.1, 36.1, and 53.8 %, respectively, among the cases and 18.2, 43.2, and 38.5 %, respectively, among the controls, which had statistical difference (*χ* ^2^ = 7.983, *P* = 0.018). The genotype frequencies of Fas −1377 also had statistical difference (*χ* ^2^ = 6.794, *P* = 0.033). In main effect analysis, FasL −844 and Fas −1377 SNPs in <60 subgroups were associated with increased risk of pulmonary AD susceptibility (Table 8).

**Table 8:** Main effects of individual risk factors on pulmonary adenocarcinoma risk by Logistic regression in age <60 years group

Variable	Crude OR (95 % CI)	*P* value	Adjusted OR (95 % CI)	*P* value
FasL −844 locus	1.612 (1.151–2.256)	0.005	1.642 (1.167–2.310)^a^	0.004
Fas −1377 locus	1.408 (1.024–1.936)	0.035	1.411 (1.020–1.951)^b^	0.038

^a^Adjusting for gender, smoking and Fas −1377 locus

^b^Adjusting for gender, smoking and FasL −844 locus

For those over age 60, there were no differences between the two groups about genotypes or alleles.

We conducted further analyses to explore whether FasL −844T/C polymorphism was associated with clinicopathological parameters in the selected population of patients (Table 9). For those <60 years, FasL −844T/C showed significant association with tumor stage (crude OR = 1.974, 95 % CI 1.113–3.502, *P* = 0.020; aOR by smoking and sex = 1.995, 95 % CI = 1.098–3.627, *P* = 0.023). For those ≥60 years, FasL −844T/C showed significant associations with stage (crude OR = 2.119, 95 % CI 1.301–3.451, *P* = 0.003; adjust OR by smoking and sex = 2.228, 95 % CI 1.351–3.675, *P* = 0.002) and lymph node metastasis (crude OR = 2.051, 95 % CI 1.252–3.361, *P* = 0.004; adjust OR by smoking and sex = 2.123, 95 % CI = 1.285–3.508, *P* = 0.003).

**Table 9:** Distribution of selected characteristics of patient subgroup cohort

	<60				≥60			
	FasL −844		Fas −1377		FasL −844		Fas −1377	
	*χ* ^2^	*P*	*χ* ^2^	*P*	*χ* ^2^	*P*	*χ* ^2^	*P*
Smoking	1.495	0.473	4.046	0.132	1.615	0.446	0.825	0.662
Gender	1.265	0.531	0.031	0.985	1.206	0.574	0.782	0.677
Tumor length	2.064	0.356	0.477	0.788	0.623	0.732	0.207	0.122
Differentiation	1.848	0.397	0.174	0.917	0.477	0.788	2.094	0.351
Visceral pleura	0.13	0.931	0.609	0.738	0.797	0.671	0.442	0.802
Stage	6.471	0.039^*^	2.069	0.355	9.594	0.008^*^	0.073	0.964
Lymph node metastasis	4.152	0.125	0.848	0.654	8.823	0.012*	0.001	1.000
Vascular tumor thrombus	3.588	0.166	4.216	0.121	0.019	0.991	2.934	0.231

*P < 0.05

## Discussion

Fas–FasL system plays an important role in regulating apoptosis and maintaining cellular homeostasis. FasL is an important gene in lung AD. Nineteen genes were designated as candidate lung tumor progression (LTP) genes because their expression changes may specially affect lung tumor progression in mice. FasL was the most important gene among these LTP genes [17].

Fas and FasL genes are located on chromosomes 10q24.1 and 1q23, respectively. Fas −1377 polymorphism is in tight linkage disequilibrium with FAS −670 polymorphisms. Fas −1377A allele disrupts Sp1 transcription factor binding sites, and FAS −670G allele abolishes STAT1-binding sites, both of which diminish Fas promoter activity and decrease gene expression [11]. FasL −844T/C polymorphism is located in the gene promoter. Higher basal expression of FasL is significantly associated with FasL −844C allele compared with FasL −844T allele. The C allele and its flanking sequence constitute CAAT box which is the binding site for CAAT Enhancer Binding Protein Beta (C/EBPb), resulting in a significantly higher basal FasL expression [16, 18]. FasL −844T/C polymorphism may influence FasL expression level and FasL-mediated signaling pathway, and ultimately, the susceptibility to cancer.

Fas–FasL system involves in immune escape with two kinds of mechanisms: FasL on tumor cells cross-links with Fas on tumor-infiltration lymphocyte (TIL) to induce TIL apoptosis [19–22]. Meanwhile, TIL in tumor microenvironment can kill each other through AICD by Fas–FasL pathway [23, 24]. Fas can regulate apoptosis effect of FasL. In our study, both FasL −844T/C and Fas −1377G/A polymorphisms were associated with the susceptibility of pulmonary AD. However, only when FasL −844 genotype was CC, Fas −1377 GG was a risk factor for lung AD, and Fas −1377 genotypes showed significant effect modification of pulmonary AD risk by FasL −844 genotype, which is consistent with the mechanism.

Sung et al. studied the association of FasL −844CC SNPs with NSCLC in Taiwan [25]. They found that the FasL −844T/C genotype was not associated with lung cancer risk in case–control study. Ter-Minassian et al. studied the association of these SNPs with NSCLC in a large case–control study in Canada [26]. No associations with NSCLC were observed in the main effects analysis for FasL −844C/T and Fas −1377G/A adjusting for age, gender, smoking status, pack-years, and years since smoking cessation. In subjects under age 60, for FasL −844C/T polymorphism, CT compared with the CC genotype was significantly associated with increased risk of NSCLC. Zhang et al. examined the contribution of these polymorphisms to risk of developing lung cancer in northern China [27]. Compared with noncarriers, there was a 1.59-fold excess risk of developing lung cancer for carriers of the Fas −1377AA genotype and 1.79-fold excess risk for carriers of FasL −844CC. Fas and FasL genotypes were determined in 582 lung cancer patients and 582 healthy control subjects who were frequency-matched for age and gender in Korea [28]. Both genotypes and Fas haplotypes exhibited no apparent relationship with the risk of lung cancer. In addition, there was no significant interaction between Fas and FasL polymorphisms in the development of lung cancer. The results suggested that Fas −1377G/A and −670A/G and FasL −844T/C polymorphisms did not significantly affect the susceptibility to lung cancer in Koreans.

We investigated the association between FasL −844T/C and Fas −1377 polymorphism on the risk of pulmonary AD in South China population. In this study, FasL −844 and Fas −1377 genotypes were risk factors for pulmonary AD. Furthermore, FASL −844CC and Fas −1377GG were risk factors for pulmonary AD by genotypes analyses. These two genotypes were risk factors for patients <60 years old and did not increase the risk of disease in patients ≥60, suggesting that patients <60 years old had strong immune function and immune suppression may increase the risk of cancer, while being ≥60 with weak immune function suffering a certain degree of immuno-suppression did not increase the risk of cancer. Fas −1377, only in the present of FasL −844CC genotype, could increase the risk of cancer. Fas −1377 genotypes showed significant effect modification of pulmonary AD risk by FasL −844 genotype by test of the interaction term adjusting for age, gender, FasL −844, and Fas −1377 SNP. This result verifies the mechanism of Fas–FasL-mediated cell apoptosis: FasL can induce cell apoptosis by cross-linking with Fas.

It had been shown that Fas expression did not correlate with age, sex, histological type, or stage of disease. However, NSCLC expressing FasL was associated with poor clinical prognosis and metastasis [25]. Sung et al. found that the FasL −844CC genotype had higher prevalence in those with advanced tumors than in those with early tumors. Not only did we find that FasL −844CC is associated with stage of pulmonary AD but also we found that this genotype is the risk factor for lymph node metastasis, especially in patients for age equal and over 60 years. So, pulmonary AD cells with FasL −844CC may have strong invasiveness.

## Conclusions

In conclusion, FasL −844 and Fas −1377 genotypes were risk factors for pulmonary AD for patients <60 old. Fas −1377, only in the present of FasL −844CC genotype, could increase the risk of cancer. Fas −1377 genotypes showed significant effect modification of pulmonary AD risk by FasL −844 genotype by test of the interaction term. FasL −844CC was a risk factor for tumor stage and lymph node metastasis. Therefore, FasL −844 genotype plays an important role in the occurrence and progression of pulmonary AD.
